# Protein engineering in Brazil: a biophysical perspective

**DOI:** 10.1007/s12551-026-01469-0

**Published:** 2026-10-05

**Authors:** Mariana Zuliani Theodoro de Lima, Gustavo Foltran Umbehaun, Atílio Tomazini

**Affiliations:** 1 Engineering School, Mackenzie Presbyterian University, Sao Paulo, Sao Paulo, Brazil; 2 OncoAI, Oncologia Inteligência Artificial, Sao Paulo, Brazil; 3 Institute of Physics, University of Sao Paulo, Sao Paulo, Brazil

**Keywords:** Protein engineering, Rational protein design, Structural biophysics, Computational protein design

## Abstract

Protein engineering has become an increasingly interdisciplinary field, integrating molecular biology, structural biology, biophysics, computational modeling, and artificial intelligence to modify protein structure, stability, dynamics, molecular recognition, and catalytic activity. This systematic review examines the historical development and scientific contributions of protein engineering research associated with Brazilian institutions between 1988 and June 2026. A total of 792 peer-reviewed articles were identified in PubMed through eight thematic search strategies covering protein and enzyme engineering, directed evolution, rational and computational design, recombinant protein expression, therapeutic proteins, structural biology, and artificial intelligence-assisted engineering. The reviewed literature reveals a transition from early residue-level mutagenesis and conventional biochemical assays to increasingly integrated experimental and computational approaches. Advances in X-ray crystallography, nuclear magnetic resonance (NMR), circular dichroism (CD), mass spectrometry, small-angle X-ray scattering (SAXS), calorimetry, molecular dynamics, and structural bioinformatics enabled the quantitative investigation of protein folding, stability, conformational landscapes, molecular interactions, and catalytic mechanisms. More recently, machine learning, protein language models, AlphaFold-derived structures, and generative approaches have expanded the capacity to predict mutations and design proteins, peptides, antibodies, and enzymes with predefined properties. Overall, protein engineering in Brazil has evolved into a mature and diverse field with applications in health, infectious diseases, agriculture, biotechnology, bioenergy, biomaterials, environmental remediation, and industrial biocatalysis. The findings indicate that biophysics has moved beyond a predominantly descriptive role and has become central to the design and validation of engineered proteins. Future progress will depend on the convergence of experimental biophysics, multiscale simulations, artificial intelligence, high-throughput validation, and stronger national and international research networks.

## Introduction

**Fig. 1:**
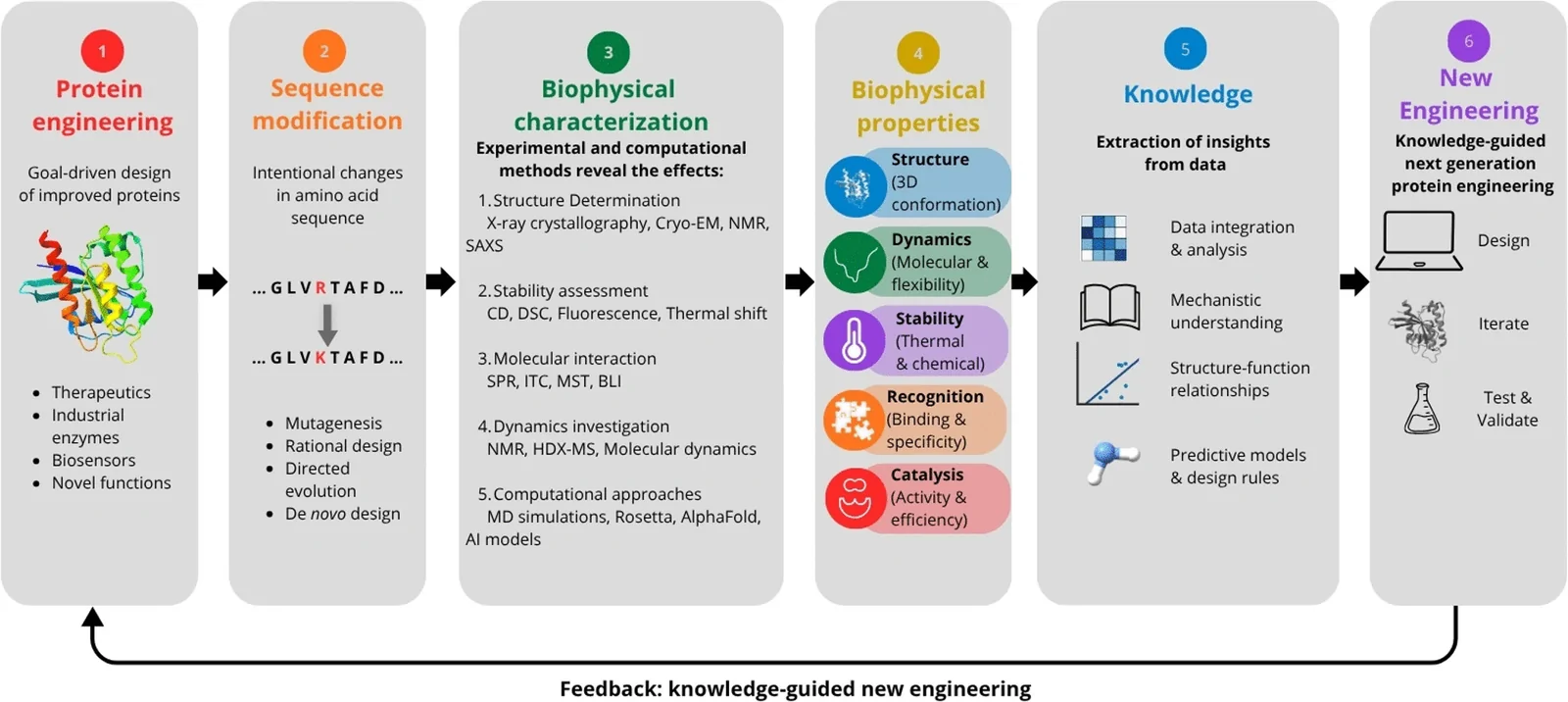
Workflow of protein engineering

To provide a comprehensive overview of protein engineering in Brazil over the past four decades, we propose to discuss the literature from a biophysical perspective, examining how biophysics has enabled or supported changes in molecular and protein properties, as well as the development of experimental and computational methods for protein characterization and their impact on the field of protein engineering (Fig. 1). This review provides the first comprehensive overview of protein engineering in Brazil. Accordingly, we aim to integrate the contributions of biophysical techniques to protein engineering.

Proteins are dynamic molecular systems whose biological functions emerge from the relationship between amino acid sequence, three-dimensional structure, conformational flexibility, stability, molecular recognition, and catalytic activity. Understanding and deliberately modifying these relationships constitute the basis of protein engineering, an interdisciplinary field that combines molecular biology, biochemistry, structural biology, biophysics, computational modeling, and, more recently, artificial intelligence. Rather than being restricted to the production of mutated proteins, contemporary protein engineering involves the rational or evolutionary exploration of sequence and conformational spaces to generate molecules with improved or entirely new properties.

The emergence of recombinant DNA technologies and site-directed mutagenesis transformed the study of proteins by allowing individual residues to be modified and their structural and functional consequences to be experimentally investigated. Early Brazilian contributions already demonstrated the value of integrating molecular engineering with biophysical measurements. Reinach and Karlsson (Reinach and Karlsson 1988), for example, used cloning, recombinant expression, and site-directed mutagenesis to investigate the role of specific residues in skeletal muscle troponin C. Subsequent studies showed that localized sequence modifications could alter calcium affinity, allosteric communication, and the stability of regulatory protein complexes (Fujimori et al. 1990; Sorenson et al. 1995). These investigations helped establish a fundamental principle of protein engineering: relatively small changes in sequence can propagate through a molecular structure and generate substantial functional effects.

During the following decades, the expansion of structural biology significantly increased the ability to characterize engineered proteins. X-ray crystallography, nuclear magnetic resonance spectroscopy, circular dichroism, fluorescence spectroscopy, mass spectrometry, small-angle X-ray scattering, calorimetry, and other biophysical techniques enabled researchers to relate sequence modifications to folding, thermodynamic stability, conformational transitions, molecular interactions, and biological activity. Structural studies of Brazilian biological systems, including toxins, enzymes, regulatory proteins, and protein–nucleic acid complexes, demonstrated that function depends not only on the presence of specific residues but also on the organization of interaction interfaces, hydration networks, oligomeric states, and flexible structural regions (Polikarpov et al. 1999; Pinheiro et al. 2003; Coltri et al. 2007).

This development was accompanied by a conceptual transition from static representations of proteins toward an ensemble-based view. Proteins are currently understood as populations of interconverting conformational states whose distributions and exchange rates influence recognition, catalysis, allostery, and adaptation. Flexible loops, linkers, terminal regions, and domain movements may control access to active sites, substrate positioning, ligand affinity, and the formation of functional complexes. Consequently, engineering a protein may require not only stabilizing a particular structure but also reshaping its energy landscape and controlling the accessibility of functionally relevant conformations.

The integration of experimental biophysics with computational approaches further expanded the scope of the field. Molecular dynamics simulations, molecular docking, quantum mechanics/molecular mechanics methods, free-energy calculations, normal-mode analysis, and structural bioinformatics made it possible to examine molecular events that are difficult to observe directly. These methods have supported the identification of stabilizing mutations, the interpretation of conformational transitions, the redesign of binding surfaces, and the investigation of catalytic mechanisms. Nevertheless, computational predictions are most informative when supported by quantitative experimental validation, since structural plausibility alone does not demonstrate improvements in affinity, selectivity, catalytic efficiency, or biological function.

Alongside rational design, directed evolution introduced an alternative strategy for navigating protein sequence space. By combining genetic diversification and experimental selection, this approach made it possible to identify beneficial mutations even when the molecular determinants of a desired property were not completely understood. Studies involving Brazilian institutions have employed directed evolution, rational mutagenesis, surface-charge engineering, domain insertion, and ancestral sequence reconstruction to improve thermostability, catalytic activity, substrate specificity, and tolerance to industrially relevant conditions (Ruller et al. 2008; Ribeiro et al. 2015; Santos et al. 2019; Martins et al. 2024). These approaches demonstrate that protein properties are interconnected: mutations introduced to enhance stability may alter flexibility or activity, while modifications intended to improve recognition may affect folding, solubility, or conformational behavior.

Molecular recognition represents another central dimension of protein engineering. Protein–protein, protein–ligand, antibody–antigen, receptor–ligand, and enzyme–substrate interactions depend on a combination of geometric complementarity, electrostatic interactions, hydrogen-bonding networks, solvent organization, interface dynamics, and conformational accessibility. Over time, research progressed from the identification of individual contact residues to the engineering of complete interfaces, antibody linkers, allosteric sites, substrate-access tunnels, and de novo binding proteins. This evolution highlights the distinction between increasing binding affinity and achieving biologically relevant selectivity or functional efficacy.

More recently, machine learning and deep learning methods have introduced a new stage in protein engineering. Predictive models can estimate the effects of mutations on stability and affinity, while generative approaches can propose sequences compatible with specific structures or molecular interfaces. Tools based on graph representations, protein language models, AlphaFold-derived structures, ProteinMPNN, and other artificial intelligence architectures are increasingly integrated with physics-based simulations and experimental biophysics (Pires, Blundell and Ascher 2016, Ribeiro-Filho et al. 2024). These advances are shifting the field from the retrospective interpretation of natural proteins toward the prospective design of proteins, peptides, antibodies, and enzymes with predefined characteristics. However, the reliability of these workflows still depends on the quality and diversity of training data, adequate sampling of conformational states, and rigorous experimental confirmation.

Brazil has actively participated in this scientific trajectory through a broad national network of universities, research institutes, and central research facilities. Key institutions, including major federal and state universities, the Oswaldo Cruz Foundation (Fiocruz), and the Brazilian Center for Research in Energy and Materials (CNPEM) with its national laboratories and synchrotron facilities, have established a robust framework for structural biology. Supported by sustained investments from national and state funding agencies, this scientific ecosystem has yielded diverse applications spanning therapeutic design, agricultural biotechnology, and industrial biocatalysis.

Despite this scientific breadth, the historical evolution, thematic organization, collaborative structure, and methodological contributions of Brazilian protein engineering have not yet been comprehensively examined from a biophysical perspective. A systematic synthesis of this production is therefore relevant for identifying how the field has evolved, which properties and biological systems have received greater attention, and how experimental and computational approaches have been integrated.

To address these questions, this review is structured around the five key biophysical properties that define protein functionality: folding, stability, dynamics, molecular recognition, and catalytic efficiency. By analyzing these universal properties through the distinct thematic lenses that define the identity of the Brazilian protein engineering community, namely, the bioprospecting of animal toxins and antimicrobial peptides, the optimization of industrial enzymes for biomass conversion, and the targeting of local and neglected pathogens, we map a clear historical progression from empirical residue-level modifications to predictive, hybrid, and AI-assisted design, providing a functional roadmap for next-generation protein engineering.

## Literature search strategy

All articles included in this review were identified through a systematic search of the PubMed database. The search strategy was organized into eight thematic blocks encompassing: (i) protein engineering, (ii) enzyme engineering, (iii) directed evolution and mutagenesis, (iv) rational and computational protein design, (v) recombinant protein expression for engineering applications, (vi) antibody and therapeutic protein engineering, (vii) structural biology applied to protein engineering, and (viii) artificial intelligence-assisted protein engineering. Each strategy combined domain-specific keywords with affiliation filters corresponding to Brazilian universities and research institutes and was restricted to publications indexed between January 1988 and June 2026. Only articles published in English were considered. Review articles, methodological protocols, communications, and letters were excluded from the analysis.

## Historical evolution of protein engineering in Brazil

**Fig. 2:**
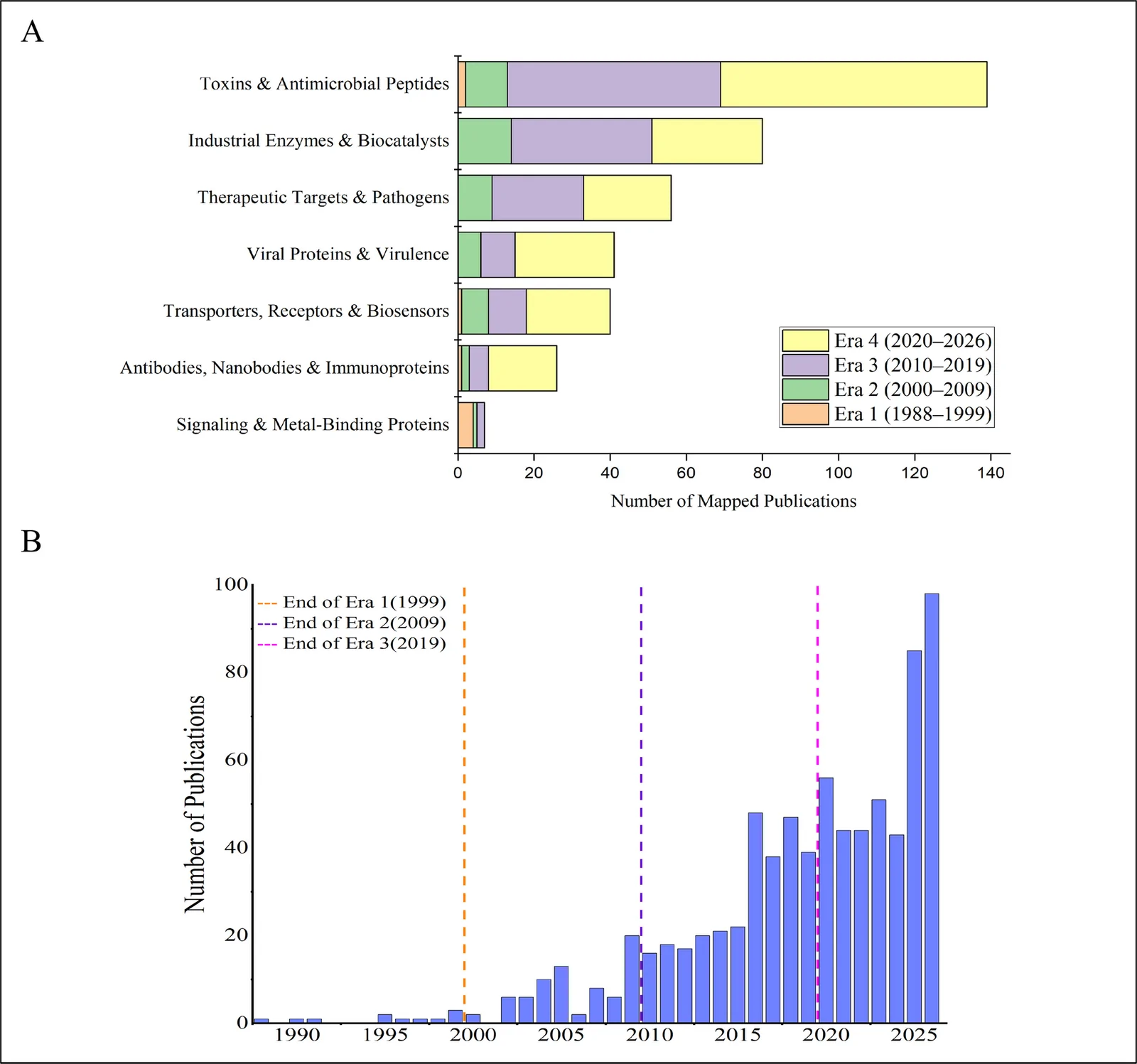
Landscape and historical evolution of protein engineering in Brazil (1988–2026). (**A**) Distribution of the major protein classes studied in Brazil. (**B**) Temporal evolution of publications in Brazilian protein engineering

Protein engineering in Brazil began with biophysical studies of model proteins, led primarily by USP and UFRJ (Reinach and Karlsson 1988; Fujimori et al. 1990; Sorenson et al. 1995). The establishment of national synchrotron facilities (CNPEM/LNLS) (Polikarpov et al. 1999; Coltri et al. 2007) and the subsequent expansion of structural biology across state and federal universities (Sperotto et al. 2004; Valadares et al. 2013) accelerated the transition from descriptive structure–function studies to mechanism-driven protein engineering. This evolution was shaped by two complementary priorities: the exploration of Brazil's biodiversity, including animal toxins, antimicrobial peptides, and plant defense proteins (Fig. 2A) (Polikarpov et al. 1999; Daniele-Silva et al. 2016; Magalhães et al. 2026), and the development of solutions for industrial biotechnology, agriculture, and infectious diseases (Pasqualoto et al. 2004; Fonseca et al. 2006). More recently, advances in structural biology, computational modeling, and artificial intelligence have enabled the rational engineering of antibodies, nanobodies, and de novo protein scaffolds, consolidating predictive and integrative approaches to protein design (Magalhães et al. 2026; Pinheiro et al. 2026).

**Fig. 3:**
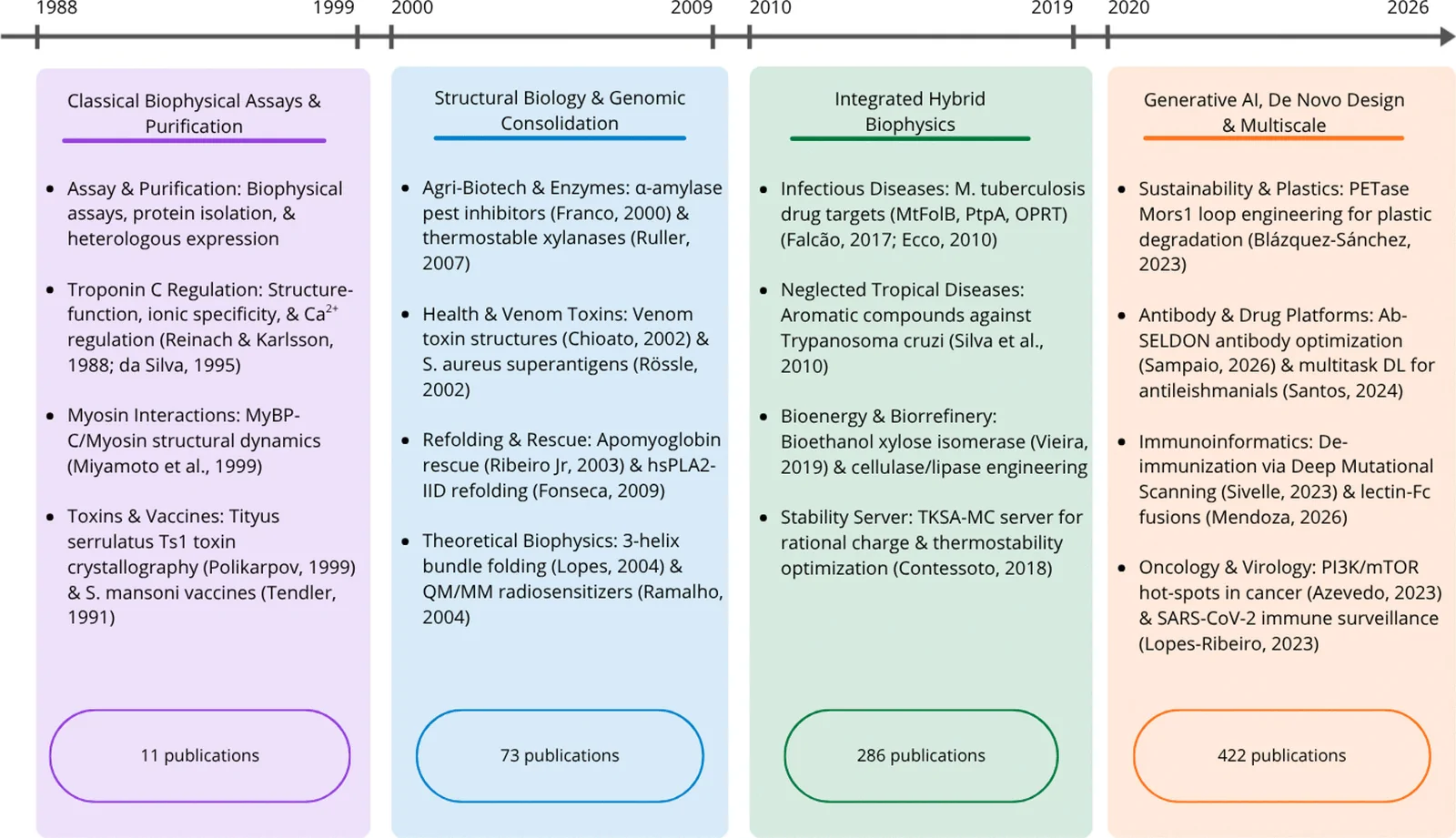
Evolution of biophysical approaches and protein engineering in Brazil (1988–2026)

To visualize this trajectory, Fig. 2B illustrates the historical evolution of protein engineering publications in Brazil from 1988 to 2026, while Fig. 3 conceptualizes this progress across four distinct methodological eras, showcasing the transition from classical biophysics to generative design. Chronologically, this sustained growth was heavily supported by targeted investments from both federal and state funding agencies.

Specifically, quantitative analysis of the surveyed literature identified the Brazilian National Council for Scientific and Technological Development (CNPq) as the leading federal funding agency, supporting 540 of the 792 publications analyzed (68.2%). At the state level, the São Paulo Research Foundation (FAPESP) was the predominant funding agency, acknowledging support in 349 publications (44.1%), through long-term funding programs such as Thematic Projects and Research, Innovation and Dissemination Centers (CEPIDs). Together, CNPq and FAPESP have played central and complementary roles in supporting the development of structural biophysics and protein engineering in Brazil.

To systematically examine the research landscape revealed by these analyses, the following sections of this review evaluate how the major protein classes identified in Fig. 2A and the publication trends shown in Fig. 2B are shaped by universal biophysical principles. Specifically, we organize this body of literature around five core functional dimensions: protein stability, folding, dynamics, molecular recognition, and catalytic efficiency. Within each dimension, we discuss how prominent biological systems in the Brazilian research landscape including disulfide-stabilized venom toxins, antimicrobial peptides, and biomass-degrading industrial enzymes have been engineered, highlighting how experimental and computational approaches evolved over time to address specific biotechnological and medical challenges.

## Engineering protein properties: a biophysical perspective

### Protein stability

An analysis of the surveyed literature reveals that approximately 57% of the articles (Table 1) address protein stability either as a primary or secondary focus, aiming to optimize protein folding, enhance thermostability, or elucidate denaturation mechanisms. The period from the beginning of this survey until 1999 marks the advent of rational mutagenesis. During this early phase, biophysical approaches were employed to investigate allosteric regulation and protein conformational changes. Protein function was evaluated using biophysical and biochemical techniques to monitor ion binding, enzymatic activity, and structural organization, including radiolabeled ion-binding assays (Reinach and Karlsson 1988; Fujimori et al. 1990), coupled ATPase activity measurements (da Silva, Kendrick-Jones and Reinach 1995), and early X-ray crystallographic analyses (Polikarpov et al. 1999).

**Table 1:** Distribution of major biophysical aspects in Brazilian protein engineering studies (N = 792)

Biophysical Aspects Addressed in Protein Engineering Studies (N = 792)	Central	Secondary	Mentioned	Not Addressed
Molecular Recognition	459 (57.9%)	260 (32.8%)	38 (4.8%)	35 (4.4%)
Catalytic Function	286 (36.1%)	147 (18.6%)	31 (3.9%)	328 (41.4%)
Protein Stability	191 (24.1%)	261 (33.0%)	124 (15.7%)	216 (27.3%)
Protein Dynamics	131 (16.5%)	301 (38.0%)	75 (9.5%)	285 (36.0%)
Protein Folding	115 (14.5%)	267 (33.7%)	66 (8.3%)	344 (43.4%)

This pioneering work by Reinach and Karlsson (Reinach and Karlsson 1988; Fujimori et al. 1990) established the first integration of site-directed mutagenesis with biophysical assays in Brazil, laying the methodological foundation for assessing protein stability. In 1995, da Silva et al. (da Silva et al. 1995) combined site-directed mutagenesis with flow dialysis and pH–stat assays to demonstrate that a single substitution in the EF-hand motif of the myosin regulatory light chain converted a mixed Ca^2^⁺/Mg^2^⁺-binding site into a Ca^2^⁺-specific site. In 1999, Polikarpov et al.(Polikarpov et al. 1999) used X-ray crystallography to determine the three-dimensional structure of the Ts1 neurotoxin from *Tityus serrulatus*, revealing a rigid disulfide-stabilized core and solvent-exposed loops that govern sodium channel recognition and toxin specificity.

The subsequent decade marked an expansion of structural biology driven by synchrotron-based X-ray crystallography, NMR, circular dichroism (CD), and high hydrostatic pressure (Chioato et al. 2002; Pinheiro et al. 2003; Ribeiro et al. 2003; Lopes et al. 2004; Coltri et al. 2007). These technologies shifted focus toward structural and packing determinants of stability. For instance, CD and functional assays identified the C-terminus of Lys49 phospholipase A₂ as a membrane-disrupting determinant independent of catalysis (Chioato et al. 2002), while atomic-resolution crystallography of neurotoxin Ts1 mapped interfacial plasticity (Pinheiro et al. 2003). NMR characterization of apo-myoglobin established how structural destabilization drives proteins toward molten globule states (Ribeiro et al. 2003), and high-pressure biophysics demonstrated that engineered internal cavities increase native-state compressibility by disrupting hydrophobic core packing (Lopes et al. 2004). Concurrently, crystallographic and modeling studies revealed how ligand-induced loop immobilization and adaptive domain rearrangements regulate stability and active-site microenvironments (Viviani et al. 2008; Coltri et al. 2007; Guzzo et al. 2009).

Between 2010 and 2019, the consolidation of hybrid biophysics integrated microsecond-scale molecular dynamics (MD) with experimental measurements, demonstrating how localized stability coordinates catalytic activity. Studies combining mutagenesis, spectroscopy, and kinetics revealed that single conservative mutations (Lorenzon et al. 2010) or post-translational modifications like S-nitrosylation (Ecco et al. 2010) restrict local loop dynamics to alter enzymatic active states. Similarly, combining solution NMR and MD simulations showed how flexible C-terminal regions govern stability and transition-state gating through transient non-covalent interactions (Sayegh et al. 2016).

In the contemporary era, the adoption of AlphaFold, SAXS, Cryo-EM, and multiscale simulations has enabled the rational engineering of stability. Recent studies demonstrate that quaternary structure modification, such as C-terminal extension-mediated oligomerization, stabilizes enzymes by reducing active-site flexibility (de Morais et al. 2020). Thermodynamic adaptation can be engineered through surface electrostatic optimization, which modulates folding energy barriers to enhance stability while preserving functional flexibility (Ferreira et al. 2020). This is complemented by algorithmic frameworks like the Proteus Search Engine to predict cooperative stabilizing mutation pairs (Barroso et al. 2020), rational hydrophobic core optimization to design stable therapeutic peptides (Almeida et al. 2020), and loop-transplantation strategies with thermophilic homologs to enhance industrial enzymes (Blázquez-Sánchez et al. 2023).

### Protein folding

Approximately 48% of the analyzed literature addresses protein folding (Table 1). The historical basis was established by Reinach and Karlsson (Reinach and Karlsson 1988) and Fujimori et al. (Fujimori et al. 1990), who pioneered the integration of site-directed mutagenesis with biophysical assays to show how localized residue interactions and long-range electrostatic networks modulate regulatory protein conformations. This was extended by Sorenson et al. (Sorenson et al. 1995), who demonstrated that coordinated interdomain communication governs the conformational stability of the troponin C complex.

The subsequent decade expanded these biophysical insights to recombinant enzymes, proving that quaternary organization and sequence-level modifications dictate folding stability. For instance, Araujo et al. (Araújo et al. 2000) resolved how subtle N-terminal modifications, such as purification tags, can introduce localized steric constraints near active sites without disrupting global fold integrity. Similarly, Fonseca et al. (Fonseca et al. 2006) established that homodimeric quaternary assembly is a strict prerequisite for both folding stability and catalytic function in metabolic enzymes. This thermodynamic understanding of folding culminated in Ruller et al. (Ruller et al. 2008), who showed that directed evolution achieves enzyme thermostability by minimizing unfolding heat-capacity changes.

During the 2010 s, hybrid approaches increasingly integrated molecular dynamics (MD) with spectroscopy and calorimetry to examine folding landscapes. Structural modeling and mutational analyses demonstrated how internal structural arrangements, such as hydrophobic ligand-binding tunnels, preserve native lipid-binding architectures and overall mitochondrial function (Busso et al. 2010). Computational design was further accelerated by combining de novo sequence generation with atomistic simulations to show that targeted sequence changes reshape the folding landscape by rebalancing structural robustness and local flexibility (Cunha et al. 2015). This led to the development of specialized computational platforms, such as TKSA-MC, which exploit charge–charge electrostatic optimizations to stabilize protein folds by reshaping their free-energy landscapes (Contessoto et al. 2018).

Since 2020, artificial intelligence and multiscale modeling have revolutionized the prediction and engineering of folding pathways. Computational pipelines have accelerated the design of therapeutic peptides by optimizing physicochemical variables to control conformational behavior and membrane activity (Quigua-Orozco et al. 2024). This AI-guided framework, combining structural predictions with energetic constraints, has successfully engineered high-affinity peptide binders (Chaves Carvalho et al. 2025) and overcome challenges in epitope accessibility for complex glycosylated targets (Costa et al. 2026).

Concurrently, the view of protein folding has transitioned from static targets to dynamic ensembles. This paradigm is exemplified by Lima et al. (Lima et al. 2026b), who integrated spectroscopy, SAXS, and multiscale MD to prove that the folding of Bothropstoxin-I depends on an equilibrium between distinct dimeric conformers, where only the extended conformation triggers functional membrane insertion. Ultimately, these contemporary studies highlight a mature field where folding is programmed and evaluated as an adaptive, multi-state landscape.

### Protein dynamics

Protein dynamics has progressively emerged as a central concept in structural biophysics and protein engineering, reflecting the recognition that protein function cannot be adequately understood from static molecular structures alone. Within the 792 documents analyzed in this systematic review, 432 studies (54.5%) (Table 1) actively addressed Protein Dynamics, including 131 articles (16.5%) in which the topic was classified as Central and 301 (38.0%) in which it was classified as Secondary. The temporal distribution of these studies reveals a marked methodological and conceptual evolution, extending from residue-level perturbation and indirect inference of conformational changes to direct measurements of molecular motion, exploration of conformational ensembles and free-energy landscapes, and, more recently, deliberate engineering of protein dynamics through computational and artificial intelligence-assisted strategies.

Early investigations approached dynamics indirectly through structural perturbations, inferring conformational behavior from functional changes. Reinach and Karlsson (Reinach and Karlsson 1988) used site-directed mutagenesis and functional assays in skeletal muscle troponin C to show that localized sequence changes propagate functional signals to distant domains. This became more explicit when Fujimori et al. (Fujimori et al. 1990) integrated mutagenesis and equilibrium dialysis to demonstrate that troponin C function relies on shifts between pre-existing conformational states. This transition from static to dynamic models was also illustrated by Viviani et al. (Viviani et al. 2008), who proved that the conformation of a flexible solvent-gating loop regulates the active-site microenvironment and emission spectrum of beetle luciferase.

By the end of the 2000 s, studies expanded from local fluctuations to large-scale transitions. Oliveira et al. (Oliveira et al. 2010) resolved an approximately 67° domain rotation in yeast thioredoxin reductase, showing that productive interactions depend on accessing specific conformational states. This framework enabled the engineering of dynamic elements; Costa and Batista (Costa, Silva and Batista 2018) designed hinge-like motions into a cellulase linker to increase conformational heterogeneity, proving that sequence variation reshapes the entire dynamic repertoire. More recently, Machado et al. (Machado et al. 2025) extended this to predictive energy landscape engineering, using “Mutational Energy Landscape Traps” to select mutations that favor predefined structural states.

The 2010 s witnessed the consolidation of hybrid biophysics, where MD simulations became computational bridges for experimental data. Figueira et al. (Figueira et al. 2011) integrated HDX-MS and MD simulations to show that agonists and antagonists redistribute the conformational populations of the thyroid hormone receptor. Similarly, Prates et al. (Prates et al. 2013) combined X-ray crystallography and MD to resolve active-site loop mobility in *Trichoderma harzianum* endoglucanase. Complementing these approaches, de Oliveira et al. (de Oliveira et al. 2015) combined SAXS and structure-based MD to demonstrate that pH-dependent conformational changes in a hyperthermostable glycoside hydrolase loop directly govern its catalytic efficiency.

High-resolution characterization of dynamics was accelerated from 2016 onward by NMR spectroscopy and microsecond-scale simulations. Sayegh et al. (Sayegh et al. 2016) used multidimensional NMR relaxation to demonstrate that the flexible C-terminus of Cdc25B phosphatase dynamically gates the active site through transient cation–π interactions. Rossi et al. (Rossi et al. 2022) combined NMR and kinetics to show that positive epistasis in ceftazidimase arises from enhanced slow-timescale backbone dynamics, proving that functional innovation can emerge from altered conformational kinetics. Furthermore, Cabrera-Muñoz et al. (Cabrera-Muñoz et al. 2019) used NMR and MD to show that the specificity of the Kazal protease inhibitor CmPI-II depends on coupled molecular motions rather than static contacts.

By the early 2020 s, protein engineering shifted entirely to ensemble-based interpretations of allostery and macromolecular phase separation. Franca de Lima et al. (Lima et al. 2021) showed that glucose-tolerance in β-glucosidases is achieved by mutations that redistribute the conformational ensemble to restrict active-site access. Ferré et al. (Ferré et al. 2023) provided a classic example of this by showing that sodium binding to an allosteric pocket in the ghrelin receptor regulates agonist affinity by shifting the conformational equilibrium towards pre-existing inactive states.

Methodological convergence has further resolved complex multi-scale architectures; Lima et al. (Lima et al. 2026b) integrated SAXS, Förster resonance energy transfer (FRET), and multiscale MD to show that the functional membrane activity of Lys49 PLA2-like toxins depends on a dynamic equilibrium between compact and extended dimeric conformations. Rosa E Silva et al. (Rosa E Silva et al. 2026) combined crystallography, NMR, and X-ray photon correlation spectroscopy (XPCS) with MD to demonstrate that chloride binding stabilizes the RNA helicase DDX3X while forming highly dynamic biomolecular condensates. This rational design paradigm was also demonstrated by Evangelista et al. (Evangelista et al. 2024), who engineered functional catalytic microreactors by fusing low-complexity domains to a chlorocatechol dioxygenase.

This shift is reflected chronologically: publications on protein dynamics grew from 29 studies (1988–2005) to 194 in the 2021–2026 period (44.9% of the total, Table 1), matching the rapid expansion of accessible high-resolution biophysical methods.

From a critical perspective, no single technique provides a complete description of protein dynamics. MD simulations remain constrained by force-field selection and sampling timescales, meaning computational trajectories cannot independently prove biological relevance without experimental cross-validation (Figueira et al. 2011; de Oliveira et al. 2015; Sayegh et al. 2016; Rossi et al. 2022; Ferré et al. 2023; Lima et al. 2026b). Conversely, experimental techniques carry their own trade-offs: SAXS provides low-resolution, ensemble-averaged shapes; HDX-MS measures protection indirectly; and NMR becomes challenging for larger molecular complexes. Therefore, robust mechanistic validation requires methodological convergence, where predicted energy landscapes are directly cross-validated with functional outcomes.

Furthermore, increased flexibility is not universally beneficial; functional efficiency depends on the precise location, timescale, and coordination of motions. Generalized mobility can compromise stability, whereas targeted rigidity can impair allosteric communication (de Oliveira et al. 2015; Lima et al. 2021). This modern view refutes deterministic allosteric pathways in favor of a population-shift model, where perturbations redistribute pre-existing conformational states (Rossi et al. 2022; Ferré et al. 2023). Consequently, the core challenge of contemporary protein engineering is to establish quantitative correspondence between predicted landscapes, experimentally observed dynamics, and measurable biological function, viewing proteins as dynamic ensembles rather than static structures.

### Molecular recognition

Molecular recognition is central to protein engineering, encompassing protein–ligand, protein–protein, antibody-antigen, receptor-ligand, and enzyme–substrate interactions. This is reflected in the analyzed dataset, where 57.9% of the studies (Table 1) address molecular recognition as a central focus. Early investigations focused on identifying structural determinants of interactions. Reinach and Karlsson (Reinach and Karlsson 1988) showed that single residue substitutions in skeletal muscle troponin C modulate allosteric information transfer and calcium binding. As crystallographic resolution advanced, studies expanded to entire interfaces. Polikarpov et al. (Polikarpov et al. 1999) identified 'Face A' of the Ts1 neurotoxin as the primary region recognizing mammalian sodium channels. Pinheiro et al. (Pinheiro et al. 2003) subsequently resolved this interaction at 1.16 Å, demonstrating that binding specificity is governed by interfacial microhydration and extensive hydrogen-bonding networks rather than simple steric complementarity.

Between 2006 and 2010, recognition became increasingly interpreted as a dynamic process. Structural flexibility and conformational adaptation were integrated into molecular targeting, as shown by Andrade et al. (Andrade et al. 2009), who used receptor-independent 4D-QSAR and MD simulations to incorporate dynamic ligand states into pharmacophore models of tuberculosis inhibitors. Similarly, Oliveira et al. (Oliveira et al. 2010) demonstrated that specific protein–protein recognition in the yeast thioredoxin system is fundamentally governed by domain-rotation dynamics, which act as a conformational prerequisite for assembling the functional complex.

During the early 2010 s, multiscale computation integrated with experimental biophysics to resolve transient energetic states and post-translational controls. Lameira et al. (Lameira et al. 2011) combined QM/MM and umbrella sampling to map the reaction coordinate of O-GlcNAcase, identifying a stepwise transition via a high-energy oxazoline intermediate. Chemical modifications were also linked to recognition; Tavares et al. (Tavares et al. 2014) showed that S-nitrosylation of AtMYB30 directly inhibits DNA binding by inducing secondary structural changes. In parallel, Moriconi et al. (Moriconi et al. 2014) combined homology modeling and docking to target a secondary allosteric pocket in C5aR, developing an inhibitor that modulates signaling without suppressing physiological recognition.

Allosteric and environmental factors were further resolved through quantitative thermodynamics and spectroscopy. Ferré et al. (Ferré et al. 2023) integrated ^23^Na-NMR, MD, and FRET binding assays to prove that sodium acts as a negative allosteric regulator of the ghrelin receptor, shifting the conformational equilibrium to favor inactive states and reduce agonist affinity. For competitive binding, Eberle et al. (Eberle et al. 2019) used isothermal titration calorimetry (ITC) and STD-NMR to differentiate and structurally interpret the competitive binding of suramin, heparin, and adenine to a bacterial DNA glycosylase.

Macromolecular architecture and spatial reorganization also govern antibody-fragment engineering without requiring direct antigen-binding site mutations. Queiroz et al. (de Queiroz et al. 2025) integrated BLI and MD to show that shortening the linker of an anti-CD22 scFv significantly enhanced binding affinity (K_D_ of 5.1 nM vs. 42.1 nM for the long linker) by stabilizing the interface. Similarly, Luz et al. (Luz et al. 2018) restored the solubility and functionality of an anti-Stx1 scFv (K_D_ of 2.26 × 10^–7^ M by SPR) simply by reversing the domain orientation (V_L_-linker-V_H_) and designing a hydrophilic linker.

Surface engineering and machine learning subsequently expanded the design of mutational spaces for affinity and selectivity. Furtado et al. (Furtado et al. 2018) combined ITC and MD to engineer a carbohydrate-binding module, achieving a 24-fold increase in selectivity for branched xyloglucan by reshaping hydrogen-bonding networks. To systematically map large mutational spaces, Pires et al. (Pires et al. 2016) introduced mCSM-lig, a graph-based machine-learning framework predicting mutational effects on ligand affinity and drug resistance, later extended to mCSM-AB for predicting antibody-antigen affinity changes (Pires and Ascher 2016).

Evolutionary reconstruction and immunoinformatics further guided sequence design. Castro-Fernandez et al. (Castro-Fernandez et al. 2017) used ancestral sequence reconstruction to show that a single E72A substitution shifted kinase specificity from glucose to fructose during natural selection. This sequence-patterning logic was applied synthetically by Porto et al. (Porto et al. 2018) via the Joker algorithm for antimicrobial peptide design. Additionally, these molecular recognition principles were extended to synthetic materials and immunotherapy: Silva et al. (Silva et al. 2017) employed density functional theory (DFT) to support the rational design of molecularly imprinted polymers as efficient adsorbents, while de Brito et al. (Brito et al. 2020) applied immunoinformatics to construct chimeric multi-epitope proteins designed to activate T-cells.

The contemporary era is defined by the integration of deep learning, generative modeling, and physics-based simulations. For interface design, Ribeiro-Filho et al. (Ribeiro-Filho et al. 2024) showed that deep learning architectures (ProteinMPNN, ESM-IF1) outperform traditional physics-based scoring in T cell receptor interface recovery. This predictive power enabled the de novo design of functional interfaces; Lima et al. (Lima et al. 2025) designed the miniprotein HB3-Core25 against the SARS-CoV-2 protease M^pro^, which was experimentally validated to bind the dimeric interface (K_D_ = 0.567 µM) and inhibit activity (IC_50_ = 0.27 µM). Similarly, Chaves Carvalho et al. (Chaves Carvalho et al. 2025) combined evolutionary algorithms, AlphaFold, and Rosetta FlexPepDock to engineer peptide inhibitors with optimized hydrophobic contacts, and Sampaio et al. (Sampaio et al. 2026) developed the automated Ab-SELDON pipeline to integrate antibody diversity data and structure predictions.

From a critical perspective, while computational modeling (docking, molecular dynamics, MM-PBSA, and AlphaFold) provides structural and thermodynamic hypotheses, robust validation requires orthogonal experimental biophysics (Eberle et al. 2019; de Queiroz et al. 2025; Luz et al. 2018; Furtado et al. 2018; Lima et al. 2025). This is especially critical because binding affinity and selectivity are distinct dimensions; a 24-fold increase in selectivity for branched over unbranched polysaccharides (Furtado et al. 2018) or allosteric modulation of affinity without modifying the binding pocket (Ferré et al. 2023) demonstrates that optimization can be directed toward selectivity rather than simple binding maximization. Furthermore, increased binding strength does not automatically ensure functional activity, highlighting the necessity of establishing direct quantitative links between physical recognition and biological inhibition (Lima et al. 2025).

Ultimately, molecular recognition has transitioned from the identification of static, isolated contacts (Reinach and Karlsson 1988; Polikarpov et al. 1999; Pinheiro et al. 2003) to the dynamic, thermodynamic engineering of complete interfaces, linker architectures, and allosteric networks (Andrade et al. 2009; Oliveira et al. 2010; Lameira et al. 2011; de Queiroz et al. 2025; Luz et al. 2018; Furtado et al. 2018). The contemporary paradigm employs hybrid workflows where deep learning and generative algorithms (Pires and Ascher 2016, Pires et al. 2016, Ribeiro-Filho et al. 2024, Sampaio et al. 2026) explore vast mutational spaces, while high-throughput and quantitative biophysical assays (Sivelle et al. 2023) provide the ultimate confirmation of functional recognition.

### Catalytic efficiency

Catalytic efficiency is a central dimension of protein engineering, addressed in 54.7% of the analyzed studies (Table 1). Early research focused on establishing causal relationships between sequence identity and molecular function. Reinach and Karlsson (Reinach and Karlsson 1988) first showed how controlled residue substitutions regulate allosteric transmission, a principle extended to ion specificity by da Silva et al. (da Silva et al. 1995), who converted a mixed Ca^2+/^Mg^2+^-binding site in myosin into a Ca^2+^-specific site via a single D12E mutation. This residue-centered approach was successfully applied to enzymatic selectivity by Marana et al. (Marana, Terra and Ferreira 2002), who identified Glu451 as a key determinant of substrate discrimination in *Spodoptera frugiperda* β-glycosidase, with the E451A substitution redirecting preference between glucose and galactose.

Between 2006 and 2010, the field transitioned from single-residue interrogations to strategies exploring broader sequence and conformational spaces. Ruller et al. (Ruller et al. 2008) used directed evolution to generate a *Bacillus subtilis* xylanase A tetra-mutant (Q7H/G13R/S22P/S179C) that shifted optimal catalytic temperature and increased T_m_ by 20 °C, linking thermostability to decreased unfolding heat capacity changes. Mechanistic studies also refined the distinction between substrate binding and turnover; Rodrigues Junior et al. (Rodrigues et al. 2009) showed that the K69A mutation in *Mycobacterium tuberculosis* shikimate dehydrogenase caused a 68-fold reduction in catalytic turnover without altering substrate dissociation, proving that affinity preservation does not guarantee catalytic competence. At a larger scale, Oliveira et al. (Oliveira et al. 2010) demonstrated that catalytic competence in the yeast thioredoxin reductase–thioredoxin system requires a 67° domain rotation, expanding enzyme optimization models from static active sites to dynamic conformational ensembles.

From 2011 onward, multiscale computational methods increasingly guided mechanistic design. Lameira et al. (Lameira et al. 2011) combined hybrid QM/MM calculations with umbrella sampling to map the reaction coordinate of O-GlcNAcase, resolving a stepwise pathway mediated by a high-energy oxazoline intermediate. Computational modeling was also incorporated to construct large chimeric architectures; Cota et al. (Cota et al. 2013) combined all-atom MD simulations with SAXS to design a functional xylanase–lichenase chimera (XylLich) for complex biomass conversion. To control activity indirectly, Ribeiro et al. (Ribeiro et al. 2015) engineered an allosterically responsive biocatalyst by inserting a xylanase domain into a xylose-binding protein, achieving a 3.5-fold increase in catalytic efficiency triggered exclusively by effector-induced conformational coupling.

Between 2016 and 2020, research emphasized enzyme performance under operationally demanding industrial environments. Alponti et al. (Alponti et al. 2016) used surface charge engineering (S22E/N32D) on a GH11 xylanase to increase thermal half-life 6.3-fold and activity 1.9-fold, demonstrating that surface modifications distant from the active site can optimize kinetics. Specificity was also engineered through evolutionary pathways; Castro-Fernandez et al. (Castro-Fernandez et al. 2017) used ancestral sequence reconstruction to identify a single E72A kinase substitution that shifted substrate specificity from glucose to fructose. Additionally, Santos et al. (Santos et al. 2019) redesigned the access tunnel of a GH1 β-glucosidase (L167W/P172L), achieving a 300% activity increase under high-glucose conditions by controlling substrate entry and product diffusion pathways outside the immediate reaction center.

The contemporary period (2021–2026) is marked by the convergence of electrostatic modeling, ancestral reconstruction, and deep learning. Contessoto et al. (de Godoi Contessoto et al. 2021) applied the TKSA-MC model to xylanase (K99E), enhancing catalytic turnover 1.27-fold while tripling its thermal half-life. AI-assisted directed evolution was introduced by Martins et al. (Martins et al. 2024), who used the inverse-folding model ESM-IF1 to prioritize a stabilizing G205K mutation in a GH62 arabinofuranosidase, yielding a 5 °C T_m_ increase and a 41.3% boost in denaturation energy. To map broader biocatalytic horizons, Paton et al. (Paton et al. 2025) developed the CATNIP platform, connecting chemical and protein sequence spaces to predict enzyme–substrate compatibility across 215 uncharacterized reactions. Finally, Lima et al. (Lima et al. 2026a) integrated ancestral reconstruction with AlphaFold3 and MD simulations to engineer the thermotolerant Rieske oxygenase AncVanA3, which achieved a 30 °C stability increase and 92% substrate conversion in aromatic O-demethylation.

Ultimately, the synthesized literature demonstrates that catalytic efficiency is a multivariable phenotype that cannot be understood through isolated active-site residues alone. Catalysis constitutes an emergent property of the entire molecular system, governed by substrate-recognition determinants (Marana et al. 2002, Castro-Fernandez et al. 2017), active-site turnover rates (Rodrigues et al. 2009), conformational transitions (Oliveira et al. 2010), transition-state energetics (Lameira et al. 2011), global architecture (Cota et al. 2013; Ribeiro et al. 2015), surface electrostatics (Alponti et al. 2016; de Godoi Contessoto et al. 2021), and access tunnels (Santos et al. 2019). While several campaigns successfully enhanced thermal stability without compromising or even while improving catalytic parameters (Ruller et al. 2008; Alponti et al. 2016; de Godoi Contessoto et al. 2021; Martins et al. 2024; Lima et al. 2026a) these traits are not interchangeable. Enhanced thermostability does not guarantee catalytic improvement, and preserving substrate binding does not ensure turnover competence (Rodrigues et al. 2009). Thus, a systematic assessment of engineered enzymes requires separate evaluation of stability, affinity, and kinetics before integrating them into a unified model.

This framework has undergone a major transition from retrospective, explanatory analyses of mutations (Marana et al. 2002, da Silva et al. 1995) to prospective, predictive methodologies. Computational modeling and quantum mechanics initially supported the description of reaction coordinates and dynamic pathways (Lameira et al. 2011; Cota et al. 2013; Ribeiro et al. 2015), whereas contemporary deep learning frameworks (ESM-IF1, CATNIP, AlphaFold3) now generate and prioritize sequence hypotheses prior to experimental work (de Godoi Contessoto et al. 2021; Martins et al. 2024; Paton et al. 2025; Lima et al. 2026a). However, predictive exploration does not replace the necessity of in vitro validation. The most robust optimization strategies rely on a reciprocal, hybrid relationship where computation accelerates sequence discovery and minimizes screening libraries, while quantitative biophysical and biochemical assays provide the ultimate confirmation of functional enhancement.

## Emerging technologies transforming protein engineering

The evolution of protein engineering in the analyzed literature reflects a transition from predominantly empirical modification of protein sequences toward an integrated framework in which sequence variation, structural dynamics, thermodynamic stability, molecular recognition, and functional activity can be quantitatively measured and increasingly predicted before experimental construction. Importantly, the impact of these emerging technologies is not demonstrated merely by the generation of new protein variants. Rather, the modifications are validated through measurable changes in biophysical properties, including thermal stability, conformational flexibility, binding affinity, oligomeric organization, catalytic activity, interaction energetics, and accessibility of functional conformational states. The progression observed in the dataset therefore indicates a gradual convergence between experimental biophysics and computational protein design.

One of the earliest transformations was produced by directed evolution, which enabled protein sequence space to be explored experimentally without requiring complete prior knowledge of the relationship between sequence and structure. A key milestone in this transition was the engineering of *Bacillus subtilis* xylanase A by Ruller et al. (Ruller et al. 2008), which demonstrated that directed evolution could achieve substantial thermostabilization and alter optimal operating temperatures. Rather than relying on sequence enrichment alone, this work established that directed-evolution campaigns in Brazil could be validated through the quantitative alteration of fundamental biophysical properties. This experimental paradigm laid the groundwork for modern hybrid approaches that integrate evolutionary screening with predictive computation**.**

This experimental paradigm remains relevant but is increasingly combined with computational prioritization. A recent example involving Brazilian and international groups integrated error-prone PCR and site-saturation mutagenesis with the inverse-folding model ESM-IF1, followed by molecular dynamics, differential scanning fluorimetry (DSF), differential scanning calorimetry (DSC), and kinetic measurements (Martins et al. 2024). The G205K mutation increased T_m_ by approximately 5 °C and denaturation energy by 41.3%, demonstrating that AI-assisted candidate selection can substantially reduce the sequence space requiring experimental screening. In this context, artificial intelligence does not replace directed evolution; rather, it changes its efficiency by prioritizing mutations predicted to be structurally compatible with the target fold and favorable for stability.

This integration illustrates a broader transformation in protein engineering: biophysical properties that were previously identified only after extensive experimental screening are increasingly becoming computational design objectives. Thermal stability, for example, can be investigated through predicted energetic compatibility, residue packing, electrostatic interactions, local flexibility, and conformational fluctuations, and subsequently validated experimentally through T_m_, DSC, DSF, circular dichroism, or activity measurements (Ruller et al. 2008; Martins et al. 2024). Similarly, binding affinity can be estimated by molecular docking, MM/PBSA or MM/GBSA calculations, structural deep learning models, or physics-based scoring functions and subsequently evaluated by techniques such as Bio-Layer Interferometry (BLI) or Microscale Thermophoresis (MST) (de Queiroz et al. 2025; Lima et al. 2025).

Molecular dynamics (MD) simulations have been particularly transformative because they add a temporal dimension to static structural models. Rather than predicting only whether a sequence can adopt a given fold, MD allows investigation of residue fluctuations, loop motions, domain rearrangements, solvent exposure, hydrogen-bond persistence, interface stability, and transitions among conformational states. Brazilian groups incorporated MD relatively early into protein-engineering workflows. Cunha et al. (Cunha et al. 2015) combined circular dichroism with MD simulations using different force fields and Rosetta-based de novo design to evaluate conformational sampling and structural stability. The study demonstrated that Rosetta could efficiently identify stability thresholds as a first computational screening step, whereas MD provided more detailed information about local instability and conformational behavior. This distinction remains important: static or scoring-based design approaches can efficiently rank candidates, while MD provides mechanistic information on whether the designed structure remains stable and how it behaves over time.

The increasing integration of MD with experimental measurements is evident in later Brazilian studies. Queiroz et al. (de Queiroz et al. 2025) investigated anti-CD22 scFv variants using MD, MM-PBSA/MM-GBSA, molecular docking, principal component analysis, BLI, flow cytometry, and fluorescence spectroscopy. Modification of linker length changed both conformational organization and experimentally measured affinity**.** In this context, MD simulations were critical to explain how linker-induced conformational flexibility dynamically reorders the binding interface, demonstrating that computational trajectories can resolve temporal effects that correlate directly with experimental avidity and membrane proximity**.** In this case, protein engineering was therefore evaluated simultaneously at the level of structure, dynamics, interaction energetics, binding affinity, and biological recognition (de Queiroz et al. 2025).

A similar integration is observed in the de novo design of the HB3-Core25 miniprotein targeting the SARS-CoV-2 main protease (Lima et al. 2025). The computational workflow represented a highly integrated predictive pipeline, combining de novo backbone generation with Rosetta-based interface design, stability screening via FoldX, MutaBind2, and Flex ddG, alongside structural validation using AlphaFold2/3 predictions, in silico alanine scanning, and molecular dynamics (MD) simulations. Importantly, this extensive multi-stage computational framework directly prioritized candidate sequences for targeted experimental testing, including recombinant expression, circular dichroism, microscale thermophoresis and enzymatic assays. The experimental validation confirmed three distinct biophysical checkpoints predicted by the integrated pipeline: exceptional structural stability (with a Tm close to 100 °C), physical interface binding, and downstream functional inhibition of the enzyme. Experimental techniques capable of characterizing protein dynamics have similarly changed the interpretation of engineered proteins. Hydrogen/deuterium exchange mass spectrometry (HDX-MS) is particularly valuable because it reports changes in solvent accessibility and conformational protection. In an early Brazilian application, Figueira et al. (Figueira et al. 2011) combined amide hydrogen/deuterium exchange, ESI–MS, MD simulations, mutagenesis, and functional assays to investigate conformational changes in the thyroid hormone receptor. Different ligands produced distinct solvent-exposure patterns, demonstrating that molecular recognition involves redistribution of conformational states. HDX therefore provides a means of experimentally assessing whether an engineered mutation or ligand modifies conformational accessibility rather than simply changing a static binding geometry.

Brazilian groups subsequently incorporated HDX-MS into more complex structural workflows. Bastos et al. (Bastos et al. 2020) combined cross-linking mass spectrometry (XL-MS), HDX-MS, I-TASSER modeling, Rosetta protein–protein docking, and GROMACS MD simulations to characterize the interaction between the natural inhibitor BJ46a and the toxin jararhagin. The resulting model identified electrostatic complementarity and hydrogen-bonding interactions at the interface. This type of workflow is important because HDX-MS and XL-MS introduce experimental structural restraints that reduce the ambiguity inherent to docking and computational modeling. Consequently, computational predictions are no longer evaluated only according to energetic scores; they can be constrained by experimentally observed accessibility and proximity information.

Small-angle X-ray scattering (SAXS) provides another complementary level of information. Unlike atomic-resolution crystallography, SAXS measures global molecular dimensions and conformational organization in solution and is therefore particularly useful for proteins displaying conformational heterogeneity or large-scale domain rearrangements. Within the analyzed literature, SAXS appears increasingly integrated with molecular simulations and other structural techniques. Its contribution to protein engineering lies primarily in testing whether a computationally proposed architecture is consistent with the ensemble of structures existing in solution. SAXS can therefore help explain changes in global shape, oligomerization, flexibility, and domain organization that may not be captured by a single static structural model.

The emergence of Cryo-EM represents a related technological advance, particularly for large protein complexes and full-length signaling proteins. However, its representation within the analyzed dataset remains substantially smaller than that of MD, SAXS, crystallography, or AI-based modeling. A study involving the Federal University of Ceará and UT Southwestern reviewed and structurally examined heme-based oxygen sensors using X-ray crystallography, cryo-EM, SAXS, LC–MS/MS, and enzymatic analysis (Gilles-Gonzalez and Sousa 2023). The work highlighted loop and oligomeric rearrangements involved in signal transduction and identified cryo-EM as particularly important for obtaining structures of full-length molecular sensors. Thus, within this dataset, cryo-EM emerges mainly as a technology capable of overcoming limitations associated with truncated crystallographic constructs and enabling characterization of large conformational assemblies (Gilles-Gonzalez and Sousa 2023). Nevertheless, its comparatively limited occurrence prevents the conclusion that cryo-EM has yet become as broadly incorporated into Brazilian protein-engineering workflows as MD or AlphaFold based modeling. While single-molecule biophysics remains a promising frontier to resolve conformational heterogeneity, its practical adoption in Brazilian protein engineering workflows remains in its infancy.

In contrast, high-throughput biophysics and mutational screening are clearly represented. High-throughput strategies increase the number of sequence variants that can be associated with experimentally measurable phenotypes and therefore help address one of the central limitations of protein engineering: the enormous size of sequence space. An illustrative example is the combination of Deep Mutational Scanning (DMS), yeast surface display, next-generation sequencing, HLA-II binding prediction, BLI, and cellular neutralization assays applied to antibody de-immunization in a collaboration involving the Butantan Institute (Sivelle et al. 2023). More than 200 variants with reduced predicted immunogenicity were identified, and selected mutations produced up to a 15-fold increase in affinity while maintaining or improving functional neutralization (Sivelle et al. 2023). High-throughput technologies therefore make it possible to map sequence–function relationships at a scale not achievable through conventional single-mutant analysis.

This approach also demonstrates how the definition of a desirable protein is expanding. Protein engineering can no longer be reduced to optimization of a single property such as affinity. High-throughput workflows can simultaneously incorporate information on binding, immunogenicity, catalytic function, stability, aggregation, or expression, thereby facilitating multi-objective optimization. The study itself identified aggregation and thermal stability as parameters that could subsequently be incorporated into the screening pipeline (Sivelle et al. 2023).

Among computational tools, Rosetta represents an important methodological bridge between classical physics-based modeling and contemporary AI-based design. In the analyzed studies, Rosetta was used for de novo sequence design, protein–protein docking, peptide docking, alanine scanning, and energetic optimization (Lima et al. 2025; Cunha et al. 2015; Bastos et al. 2020). Its major contribution is the ability to estimate whether proposed structural modifications are compatible with favorable molecular interactions and energetics. Rosetta can therefore support optimization of protein stability, interface packing, hydrogen bonding, electrostatic complementarity, and sequence–structure compatibility. However, its scoring functions remain approximate, and several studies in the dataset explicitly use subsequent MD or experimental validation to evaluate predictions (Lima et al. 2025; Cunha et al. 2015; Ribeiro-Filho et al. 2024).

The introduction of AlphaFold and related structural prediction models produced a further change because high-quality structural hypotheses could be generated from sequence at unprecedented scale. Brazilian groups have rapidly incorporated AlphaFold2 and AlphaFold3 into workflows involving enzymes, peptides, antibodies, receptors, and de novo proteins. The technology is particularly useful for predicting overall fold, domain arrangement, structural compatibility of engineered sequences, and in some cases molecular complexes. However, AlphaFold predictions do not directly provide experimentally validated thermodynamic stability, binding affinity, catalytic rate, or conformational kinetics.

For this reason, the most informative studies in the dataset combine AlphaFold with complementary methods. While AlphaFold revolutionized the forward problem of predicting three-dimensional structures from amino acid sequences, inverse folding architectures like ProteinMPNN and ESM-IF1 inverted this design paradigm, allowing researchers to generate novel sequences tailored to fit and stabilize predefined structural backbones. This transition from structure prediction to sequence generation is well-illustrated in the study of T cell receptor design conducted by Brazilian groups at LNBio/CNPEM and UNICAMP in collaboration with US researchers (Ribeiro-Filho et al. 2024). The workflow combined ProteinMPNN, ESM-IF1, Rosetta Design, TCRModel2/AlphaFold2, MD, MM/PBSA, and alanine scanning. ProteinMPNN and ESM-IF1 outperformed the physics-based Rosetta approach in recovering native TCR interface sequences while maintaining comparable predicted stability and affinity (Ribeiro-Filho et al. 2024). These results illustrate a major transformation in protein engineering: deep learning models can now infer which amino-acid sequences are structurally compatible with a predefined backbone or interface.

ProteinMPNN is therefore particularly relevant to inverse protein folding. Rather than asking which structure a sequence will adopt, inverse-folding models ask which sequences are compatible with a target structure. The relevant biophysical properties addressed indirectly include structural compatibility, interface organization, residue packing, and maintenance of stable interactions. Yet these models do not independently establish kinetic or thermodynamic performance, reinforcing the need for MD and experimental affinity or stability measurements (Ribeiro-Filho et al. 2024). Looking forward, the integration of de novo backbone generation platforms like RFdiffusion represents the next logical leap, expanding protein design from sequence optimization on fixed native scaffolds toward the generation of entirely novel, non-natural structural architectures.

Moving beyond structure-conditioned sequence design, conditional Protein Language Models (PLMs) shift the design objective toward native evolutionary sequence probability. These models learn statistical relationships among amino-acid sequences from large protein datasets and can subsequently be adapted for prediction or generation. The study Fine-tuning of conditional Transformers improves in silico enzyme prediction and generation combined conditional Transformers based on ProGen with ESMFold for structural assessment, CLEAN for functional prediction, and UniKP for kinetic prediction (Nicolini et al. 2025). The resulting framework generated enzyme sequences predicted to preserve three-dimensional integrity and functional characteristics. PLMs therefore extend the engineering objective beyond structural compatibility to include predicted biochemical function and, through auxiliary models, kinetic properties.

The properties accessible to these emerging computational approaches differ substantially. AlphaFold predominantly addresses structure; ProteinMPNN and ESM-IF1 address sequence–structure compatibility; Rosetta evaluates energetic and structural feasibility; MD explores conformational stability and dynamics; MM/PBSA and related methods estimate interaction energetics; PLMs explore sequence probability, functional relationships, and sequence generation. Their greatest potential emerges when these approaches are integrated rather than interpreted independently.

This integration is exemplified by the AI-guided peptide design pipeline developed by Chaves Carvalho et al. (Chaves Carvalho et al. 2025), which combined genetic algorithms with AlphaFold2, Rosetta FlexPepDock, and GROMACS MD to engineer high-affinity SARS-CoV-2 Mpro inhibitors. By evaluating sequence space, structural configurations, and relative binding energetics in a unified computational loop, such workflows showcase the predictive potential of combining evolutionary algorithms with structural deep learning to generate optimized structural hypotheses prior to downstream in vitro validation.

The Brazilian trajectory represented in the dataset is particularly relevant because it shows that incorporation of emerging technologies did not occur only at the most recent AI stage. Brazilian groups participated in successive technological transitions. In 2007, researchers were already using directed evolution together with thermodynamic characterization to engineer thermostability (Ruller et al. 2008). By 2011, groups from USP, UNESP, UNICAMP, and LNBio were combining HDX-based mass spectrometry with MD to investigate conformational states (Figueira et al. 2011). During the following decade, Fiocruz, Butantan, CNPEM/LNBio, UNICAMP, UFPE, UFC, UFMG, UFSJ, and other institutions progressively incorporated Rosetta, MD, structural mass spectrometry, high-throughput screening, AlphaFold, inverse-folding models, deep learning methods, and AI-guided sequence optimization (de Queiroz et al. 2025; Lima et al. 2025; Bastos et al. 2020; Sivelle et al. 2023; Ribeiro-Filho et al. 2024; Chaves Carvalho et al. 2025).

This temporal progression demonstrates that Brazilian research groups have transitioned through successive technological eras in step with the international community, establishing a robust culture of hybrid experimental-computational workflows. In these pipelines, molecular dynamics and machine learning predictions do not operate in isolation; instead, they serve to compress search spaces and prioritize candidates, while experimental techniques (such as CD, SAXS, HDX-MS, and kinetics) provide the ultimate thermodynamic and functional validation.

From a critical perspective, the principal transformation produced by emerging technologies is therefore not simply increased computational power. It is the progressive conversion of protein engineering into a quantitative, multi-property design problem. Earlier workflows generally generated variants first and characterized their properties afterward. Contemporary approaches increasingly define desired properties, such as higher thermal stability, reduced conformational flexibility in a specific region, stronger interface affinity, altered specificity, enhanced catalytic activity, or stabilization of a selected conformational state and use computational methods to identify candidate sequences expected to satisfy these constraints.

Nevertheless, each technology accesses only part of the underlying biophysical problem. Cryo-EM and SAXS provide structural information at different resolutions; HDX-MS reports changes in accessibility and conformational protection; BLI and MST quantify binding; DSF and DSC characterize thermal stability; MD describes molecular motions and conformational persistence; Rosetta evaluates structural and energetic compatibility; AlphaFold predicts structural arrangements; ProteinMPNN and inverse-folding models propose compatible sequences; and PLMs can generate sequences conditioned on learned relationships between sequence and function. No individual approach simultaneously determines structure, thermodynamic stability, conformational dynamics, affinity, kinetics, specificity, and biological activity.

Consequently, the most convincing evidence in the analyzed literature arises from orthogonal validation. A computationally designed sequence gains substantially greater credibility when AlphaFold or Rosetta structural predictions are consistent with MD stability, when predicted binding is confirmed by BLI or MST, when predicted thermostability is confirmed by DSF/DSC or CD, and when these physical improvements result in measurable catalytic or biological function (Martins et al. 2024; de Queiroz et al. 2025; Lima et al. 2025; Ribeiro-Filho et al. 2024). This convergence is essential because computational models can generate plausible molecular solutions that are not necessarily experimentally realizable.

Overall, emerging technologies are reshaping protein engineering through three interconnected transformations. First, they expand the accessible sequence and structural space through directed evolution, high-throughput screening, deep mutational scanning, and generative computational models. Second, they allow an increasing number of biophysical properties including fold compatibility, thermostability, flexibility, conformational dynamics, interface organization, binding energetics, specificity, and catalytic potential to be predicted or optimized before extensive experimental screening. Third, the integration of advanced biophysical measurements with AI-assisted design is transforming protein engineering from a predominantly trial and error discipline into an iterative design–predict–measure–refine process. The Brazilian studies represented in this systematic review demonstrate progressive incorporation of this paradigm, evolving from directed evolution and thermodynamic characterization to hybrid workflows integrating MD, Rosetta, structural mass spectrometry, AlphaFold, ProteinMPNN, Protein Language Models, and experimental biophysical validation (Fig. 3).

## Conclusion

This systematic review demonstrates that protein engineering in Brazil has evolved from early studies based on site-directed mutagenesis and conventional biochemical assays toward an increasingly integrated and predictive field. Over the past four decades, advances in experimental and computational biophysics have enabled a deeper understanding and deliberate modification of protein folding, stability, dynamics, molecular recognition, and catalytic activity.

In this trajectory, biophysics has moved beyond its traditional role as a characterization tool and has become central to the design process itself, guiding mutation selection, structural optimization, interface engineering, and functional validation. The literature also shows that protein properties are interdependent, requiring combined structural, thermodynamic, dynamic, and functional analyses to ensure that improvements in one characteristic do not compromise others.

More recently, artificial intelligence, molecular simulations, and generative models have expanded the capacity to predict and design proteins with predefined properties. Rather than replacing in vitro screening, these predictive models act as search-space compressors that prioritize high-probability candidates, establishing a reciprocal, hybrid relationship where structural predictions and physics-based simulations are systematically validated by quantitative biophysical measurements.

Overall, the convergence between biophysics, artificial intelligence, and protein engineering represents the main opportunity for the future development of the field in Brazil. Strengthening national research networks, expanding access to advanced infrastructure, and promoting interdisciplinary training will be essential for transforming scientific knowledge into innovative solutions for health, biotechnology, agriculture, sustainability, and industry.

## Data Availability

No datasets were generated or analysed during the current study.
